# Topological Organization of Functional Brain Networks in Healthy Children: Differences in Relation to Age, Sex, and Intelligence

**DOI:** 10.1371/journal.pone.0055347

**Published:** 2013-02-04

**Authors:** Kai Wu, Yasuyuki Taki, Kazunori Sato, Hiroshi Hashizume, Yuko Sassa, Hikaru Takeuchi, Benjamin Thyreau, Yong He, Alan C. Evans, Xiaobo Li, Ryuta Kawashima, Hiroshi Fukuda

**Affiliations:** 1 Department of Nuclear Medicine and Radiology, Institute of Development, Aging and Cancer, Tohoku University, Sendai, Japan; 2 Division of Developmental Cognitive Neuroscience, Institute of Development, Aging and Cancer, Tohoku University, Sendai, Japan; 3 Smart Ageing International Research Centre, Institute of Development, Aging and Cancer, Tohoku University, Sendai, Japan; 4 Department of Functional Brain Imaging, Institute of Development, Aging and Cancer, Tohoku University, Sendai, Japan; 5 Department of Biomedical Engineering, School of Materials Science and Engineering, South China University of Technology, Guangzhou, China; 6 State Key Laboratory of Cognitive Neuroscience and Learning, Beijing Normal University, Beijing, China; 7 McConnell Brain Imaging Centre, Montreal Neurological Institute, McGill University, Montreal, Quebec, Canada; 8 Department of Radiology, Albert Einstein College of Medicine, Yeshiva University, Bronx, New York, United States of America; Institute of Psychology, Chinese Academy of Sciences, China

## Abstract

Recent studies have demonstrated developmental changes of functional brain networks derived from functional connectivity using graph theoretical analysis, which has been rapidly translated to studies of brain network organization. However, little is known about sex- and IQ-related differences in the topological organization of functional brain networks during development. In this study, resting-state fMRI (rs-fMRI) was used to map the functional brain networks in 51 healthy children. We then investigated the effects of age, sex, and IQ on economic small-world properties and regional nodal properties of the functional brain networks. At a global level of whole networks, we found significant age-related increases in the small-worldness and local efficiency, significant higher values of the global efficiency in boys compared with girls, and no significant IQ-related difference. Age-related increases in the regional nodal properties were found predominately in the frontal brain regions, whereas the parietal, temporal, and occipital brain regions showed age-related decreases. Significant sex-related differences in the regional nodal properties were found in various brain regions, primarily related to the default mode, language, and vision systems. Positive correlations between IQ and the regional nodal properties were found in several brain regions related to the attention system, whereas negative correlations were found in various brain regions primarily involved in the default mode, emotion, and language systems. Together, our findings of the network topology of the functional brain networks in healthy children and its relationship with age, sex, and IQ bring new insights into the understanding of brain maturation and cognitive development during childhood and adolescence.

## Introduction

Recent developments in generating a network map of the human brain, known as the human connectome, provide new insights into the organization of the brain's structural connections and their role in shaping functional dynamics [1], [2]. The features of the structural and functional networks in the human brain have been well defined, such as small-world topology, highly connected hubs, and modularity [3], [4], [5]. Great efforts in the study of the human connectome have greatly expanded our knowledge of the topological principles of brain network organization in the healthy, developing, aging, and diseased brains [6], [7]. Developmental changes in the functional brain networks include two general principles: 1) regional interactions change from being predominately anatomically local in children to interactions spanning longer cortical distances in young adults and 2) this developmental change in functional connectivity occurs via mechanisms of segregation of local regions and integration of distant regions into disparate subnetworks [8].

However, the sex- and intelligence-related differences in the functional brain networks in children remain largely unknown. Several previous studies have indicated significant sex differences in the network properties of structural [9], [10] and functional brain networks [11], [12], [13], [14] in adults. There is also evidence that intelligence is associated with the topological organization of structural [15] and functional brain networks [16] in adults. Therefore, we hypothesized that 1) the developmental trajectories of the functional brain networks might be affected by sex and 2) intelligence quotient (IQ) might have a significant effect on functional brain networks in healthy children.

In this study, we used resting-state fMRI (rs-fMRI) to investigate the effects of age, sex, and IQ on the organizational patterns of functional brain networks in healthy children. Rs-fMRI has emerged as a novel method to assess the spontaneous or intrinsic activity of the brain and to study developmental changes in the functional interactions between brain regions [17], [18]. We measured functional connectivity [19] by calculating the correlations between the time series of any pair of the 90 regions in the whole brain (defined by a prior atlas) during the resting state. A correlation matrix was obtained in each of 51 healthy children aged from 6 to 18 years and further thresholded into a binary, undirected network underlying the topological organization of a functional brain network. Finally, we investigated the effects of age, sex, and IQ on the network properties at both the global and regional levels.

## Materials and Methods

### Ethics Statement

Written informed consent was obtained from each subject and his/her parent after the receipt of a full explanation of the purpose and procedures of the study, according to the Declaration of Helsinki (1991), prior to MR image scanning. Approval for these experiments was obtained from the ethics committee of Tohoku University School of Medicine.

### Subjects

We collected brain MR images from 291 subjects (146 boys, 145 girls; age range, 5.6–18.4 years) who were recruited from various kindergartens, elementary schools, junior high schools, and high schools in Miyagi Prefecture in Japan [20], [21], [22], [23], [24]. Briefly, all subjects were healthy children without any history of neurological or psychiatric disorders. We announced that only right-handed children can participate in this study in an advertisement used in the subject recruitment and also confirmed that all subjects were right-handed using the self-writing questionnaire “Edinburgh Handedness Inventory” [25].

In this study, we acquired the rs-fMRI and IQ data from a subset of all subjects, including 60 healthy Japanese children (24 boys, 36 girls; 5.7–18.4 years). Trained examiners collected IQs from subjects over the age of 16 years by administering the Japanese version of the Wechsler Adult Intelligence Scale (WAIS), 3rd edition [26]. For subjects younger than 16 years of age, the Japanese version of the Wechsler Intelligence Scale for Children (WISC), 3rd edition [27] was used. Full-scale IQs from the score of the WAIS/WISC for each subject were calculated.

### Image acquisition and preprocessing

All images were collected using a 3-T Philips Intera Achieva scanner. A total of 34 transaxial gradient-echo images (64×64 matrix, TR = 2000 msec, TE = 30 msec, flip angle = 70°, FOV = 24 cm, 3.75 mm slice thickness-3.75*3.75*3.75 voxels) covering the entire brain were acquired using an echo planar sequence. For this scan, 160 functional volumes were obtained. The subjects were instructed to keep their eyes closed, relax their minds, and remain motionless as much as possible during the EPI data acquisition.

For each subject, the first ten volumes were discarded to allow for T1 equilibration effects and the adaptation of the subjects to the circumstances, leaving 150 volumes for further analysis. Image preprocessing was carried out using the SPM5 package (http://www.fil.ion.ucl.ac.uk/spm) and Data Processing Assistant for Resting-State fMRI (DPARSF) [28]. First, all functional images were corrected for the acquisition time delay between the slices of each volume using the sinc interpolation and for the geometrical displacement due to head movement using a six-parameter (rigid body) spatial transformation [29]. Nine subjects (6 boys/3 girls) were excluded according to the criteria that head motion was less than 3 mm of displacement or 3 degrees of rotation in any direction. After the correction, the images of 51 subjects (18 boys/33 girls) were normalized to the stereotaxic space [30] using an optimum 12-parameter affine transformation and nonlinear deformations [31] and then resampled to 3-mm isotropic voxels. Finally, the resulting data were further temporally band-pass filtered (0.01–0.1 Hz) to reduce the effects of low-frequency drift and high-frequency physiological noises.

### Construction of functional brain networks

To construct a functional brain network, we employed an automated anatomical labeling (AAL) atlas [32] to parcellate the whole brain into 90 regions (45 in each hemisphere, *N* = 90). The names of the 90 regions and their corresponding abbreviations are listed in Table S1. The mean time series of each region was then acquired by averaging the time series of all voxels within that region. Several sources of spurious variances arising from the estimated head-motion profiles, white matter signals, and whole brain signals were further removed by multiple linear regression analysis [33], [34]. The residual of this regression was then used to substitute for the raw mean time series of the corresponding regions. The functional connectivity between a pair of regions was defined as the Pearson's correlation coefficient in the residual time courses. Thus, a functional connectivity matrix (or correlation matrix) (*r_ij_*, *N*×*N*) can be obtained for each subject. Each functional connectivity matrix can be converted to a binary, undirected network *G* using a cost threshold (*t*, 0<*t*<1), which is equivalent to the ratio between the number of edges and all possible edges [35].
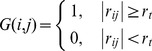
In this study, we first applied a range of cost threshold (0.05≤*t*≤0.5, step = 0.01) to investigate the network properties. Such a thresholding approach can normalize all networks to have the same number of edges or wiring cost and thus provide an avenue to detect age-related changes in the topological organization [36], [37]. Finally, we adopted the following complementary approaches to select the small-world regime as a range of cost threshold (0.2≤*t*≤0.35): (1) the average of the number of connections over all nodes is larger than the log of the number of nodes (*N* = 90) ensuring that the small-world properties are estimable [38] and (2) the resulting brain networks are sparse but fully connected and have distinguishable properties in comparison with the degree-matched random and regular networks, respectively [39], [40], [41]. To confirm our results, we also repeated all analyses using weighted, undirected network (see Text S1) and found similar results in both global network properties and regional nodal properties (see Text S2, Table S3, S4, S5, S6, S7, S8, S9).

### Network analysis

Five small-world parameters (clustering coefficient, characteristic path length, normalized clustering coefficient, normalized characteristic path length, and small-worldness) and two efficiency parameters (local efficiency and global efficiency) were computed to characterize the global topological organization of the functional brain networks. Three regional nodal parameters (node degree, node efficiency, and node betweenness) were used to examine the properties of the 90 brain regions. Here, the global network parameters and the regional nodal parameters were briefly described as follows [42] and calculated using the Brain Connectivity Toolbox (www.brain-connectivity-toolbox.net). BrainNet Viewer (http://www.nitrc.org/projects/bnv/) was used for visualization of regional nodal properties in anatomical space.

#### Global network parameters

For a graph *G* with *N* nodes and *K* edges, the clustering coefficient, *C*, of the graph *G* is the average of the clustering coefficient over all nodes [38]:
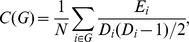
where *D_i_* is the number of edges connected to the node *i* and *E_i_* is the number of edges in the subgraph including the neighbors of node *i*. The characteristic path length, *L*, of the graph *G* is defined as [43]:
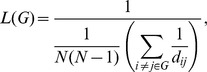
where *d_ij_* is the shortest path length between nodes *i* and *j*. The normalized clustering coefficient, *NC* = *C/C_rand_*, and the normalized characteristic path length, *NL = L/L_rand_*, were also computed, where *C_rand_* and *L_rand_* are, respectively, the mean clustering coefficient and the mean characteristic path length of 1000 matched random networks that preserve the same number of nodes, edges, and degree distribution as the real networks [44]. The small-worldness, *SW*, of the graph *G* is computed as the ratio between *NC* and *NL*, *SW = NC/NL*. The network topology may be said to correspond to a “small world” if *NC*>1 and *NL*≈1 [38] or if *SW*>1 [45]. In addition to the conventional small-world parameters (*C* and *L*), more biologically sensible properties of the brain networks are the efficiency parameters (global efficiency [*GE*] and local efficiency [*LE*]), which measure the capability of the network with regard to information transmission at the global and local levels, respectively [35]. The global efficiency of the graph *G* can be computed as:
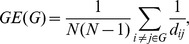
where *d_ij_* is the shortest path length between nodes *i* and *j*. The local efficiency of the graph *G* is defined as:
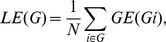
where *GE(G_i_)* is the global efficiency of *G_i_*, the subgraph of the neighbors of node *i*.

#### Regional nodal parameters

The node degree, *ND*, of a node is the number of connections that link it to the rest of the network. It is the most fundamental network measure, and most other measures are ultimately linked to it. The node efficiency, *NE*, for a given node *i* is defined as the inverse of the mean harmonic shortest path length between this node and all other nodes in the network [35], [36]:
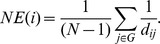
It measures the ability of a node to propagate information with the other nodes in a network. The node betweenness, *NB*, of a given node *i* is defined as [46]:
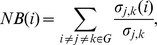
where *σ_j,k_* is the number of shortest geodesic paths between nodes *j* and *k*, and *σ_j,k_(i)* is the number of shortest geodesic paths between nodes *j* and *m*, which pass through node *i*. It captures the influence that one node has over the flow of information between all other nodes in the network. In this study, we normalized the regional nodal parameters of a node by the average value of all nodes. A node with high value (>mean + SD) in any of the regional nodal parameters was considered as a hub in the network [47], [48].

### Statistical analysis

With regard to the functional brain network in each subject, we averaged the global network parameters and the regional nodal parameters over the small-world regime (0.2≤*t*≤0.35) as the summary network parameters [39]. We applied a general linear model (GLM) to analyze the effects of age, sex, and their interaction on the summary network parameters. To detect the development trajectories of the linear and quadratic age-related changes in each summary network parameter (indicated as *Y* in the following equations), we used two multiple linear regressions (Model I and II) that modeled mean value, age, and age^2^ as predictors, with sex as a covariate. We then determined the best model among the two regressions based on Akaike's information criterion (AIC) [49].
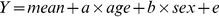
(I)

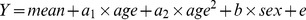
(II)To detect the sex-related difference in each summary network parameter and its development, we performed another multiple linear regression analysis (Model III), which included age, sex, and age-by-sex interaction, to examine both positive (male>female) and negative (male<female) contrasts as well as positive and negative age-by-sex interactions.
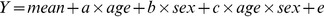
(III)If a significant age-by-sex interaction were found, a Pearson's correlation analysis was further performed between age and the summary network parameter in each sex group. For the analysis of the IQ-related difference, we first applied a multiple linear regression (Model III) on both IQ and each summary network parameter to model the effects of age, sex, and age-by-sex interaction; the residuals of the regression were then used to calculate the Pearson's correlation between IQ and the summary network parameters [50]. For all the analysis, the significance levels (*p*-values) were provided in two categories (*p*<0.05 and *p*<0.01).

## Results

### Economic small-world organization

**Figure 1:**
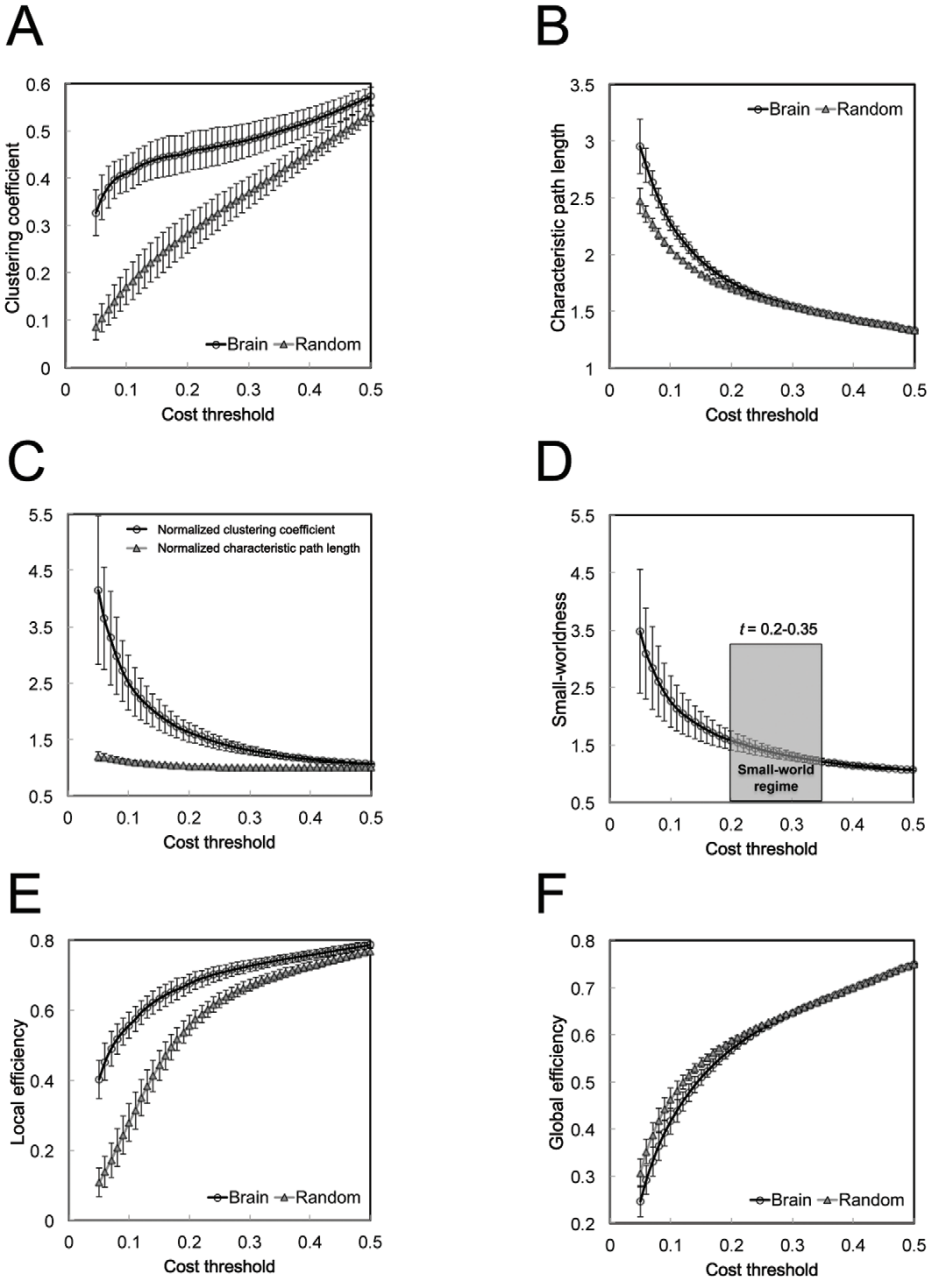
Global network properties. (A) The clustering coefficient and (B) the characteristic path length are shown as a function of cost thresholds and compared to the matched random networks. (C) The normalized clustering coefficient, the normalized characteristic path length, and (D) the small-worldness are shown as a function of cost thresholds. Note that the small-world regime of cost threshold adopted in this study was from 0.2 to 0.35. (E) The local efficiency and (F) the global efficiency are shown as a function of cost thresholds and compared to the matched random networks. Error bars indicate standard error in all subjects.

At a global level, some key organizational properties (e.g., clustering coefficient, characteristic path length, local efficiency, and global efficiency) of the functional brain networks were demonstrated as a function of the cost threshold (0.05≤*t*≤0.5) (Figure 1). The functional brain networks in healthy children showed a much higher clustering coefficient but similar characteristic path length compared with the matched random networks (Figure 1 A and B). The normalized clustering coefficient was much larger than 1, whereas the normalized characteristic path length was similar to 1 (Figure 1C). The small-worldness was larger than 1.2 over the small-world regime (0.2≤*t*≤0.35) (Figure 1D). Moreover, all functional brain networks demonstrated a higher fault tolerance of local efficiency (Figure 1E) but an approximately equivalent parallel information processing of global efficiency (Figure 1F) compared with the matched random networks.

**Figure 2:**
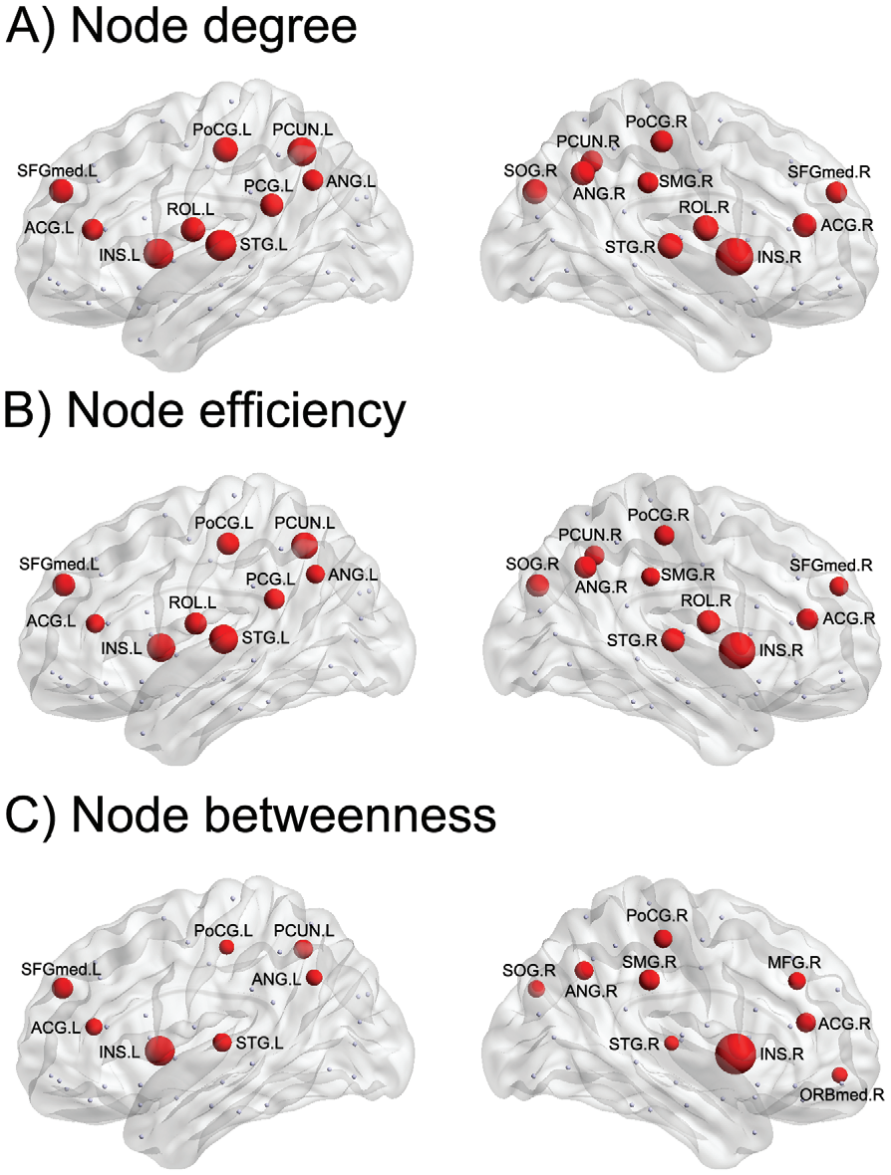
Global hubs in functional brain networks. Global hubs are defined as the brain regions with higher values (>Mean + SD) in any of (A) node degree, (B) node efficiency, and (C) node betweenness. The global hubs are shown in red with node sizes that indicate the values in regional nodal parameters. For a description of the abbreviations, see Table S1.

At the regional level, we defined 21 global hubs as the brain regions with higher values (>mean + SD) in any of the regional nodal parameters across all subjects (Figure 2, see Table S2). In particular, 14 of the 21 global hubs (the bilateral anterior cingulate gyrus [ACG], bilateral superior temporal gyrus [STG], bilateral angular gyrus [ANG], bilateral postcentral gyrus [PoCG], bilateral insula [INS], left precuneus [PCUN], left medial superior frontal gyrus [SFGmed], right superior occipital gyrus [SOG], and right supramarginal gyrus [SMG]) were identified by all of the regional nodal parameters. Five global hubs (the bilateral rolandic operculum [ROL], left posterior cingulate gyrus [PCG], right SFGmed, and right PCUN) were identified by both the node degree and node efficiency. Two global hubs (the right middle frontal gyrus [MFG] and right medial orbitofrontal cortex [ORBmed]) were identified only by the node betweenness. These global hubs were mainly composed of recently evolved association and primitive paralimbic regions [51].

### Effects of age and sex on global network properties

We analyzed the effects of age, sex, and age-by-sex interaction on the summary global network parameters (Table 1). Linear positive age-related changes (*p*<0.05) were found in the normalized clustering coefficient, small-worldness, and local efficiency. There was no significant age effect on the clustering coefficient, characteristic path length, normalized characteristic path length, and global efficiency. We also found significant sex differences (*p*<0.05) in 3 global network parameters in which the girls showed significantly higher values in the characteristic path length and normalized characteristic path length but significantly lower values in the global efficiency compared with the boys. There was no significant age-by-sex interaction in all global network parameters.

**Table 1:** The effects of age, sex, and IQ on global network properties.

	Age effect^a^			Sex effect^b^			Age-sex interaction^b^		IQ effect^c^	
	T-score	*p*-value	Model	T-score	*p*-value	Model	T-score	*p*-value	*r*	*p*-value
*C*	1.491	0.142		−1.970	0.055		−0.484	0.630	−0.126	0.380
*L*	0.365	0.717		**−2.295**	**0.026**	**F>M**	−0.106	0.916	−0.100	0.487
*NC*	**2.254**	**0.029**	**L+**	0.037	0.971		1.259	0.214	0.064	0.657
*NL*	0.459	0.648		**−2.194**	**0.033**	**F>M**	0.112	0.911	−0.106	0.458
*SW*	**2.170**	**0.035**	**L+**	0.180	0.858		1.230	0.225	0.069	0.630
*LE*	**2.297**	**0.026**	**L+**	−1.734	0.089		−0.421	0.676	−0.093	0.516
*GE*	−0.355	0.724		**2.285**	**0.027**	**F<M**	0.110	0.913	0.075	0.602

a Two multiply linear regressions that modeled age and age^2^ as predictors, along with sex as a covariate; the best model was determined by AIC.

b A multiply linear regression that modeled age, sex, and age-sex interaction.

c Pearson's correlation analysis between IQ and global network properties, each of which was regressed by a multiply linear regression that modeled age, sex, and age-sex interaction.

L+: Linear regression model showing significant positive correlation. F>M: female shows significantly higher values than male; F<M: female shows significantly lower values than male. Significances are set at *p*<0.05 and shown by bold characters.

*C*, clustering coefficient; *L*, characteristic path length; *NC*, normalized clustering coefficient; *NL*, normalized characteristic path length; *SW*, small-worldness; *LE*, local efficiency; *GE*, global efficiency.

### Effects of age and sex on regional nodal properties

**Figure 3:**
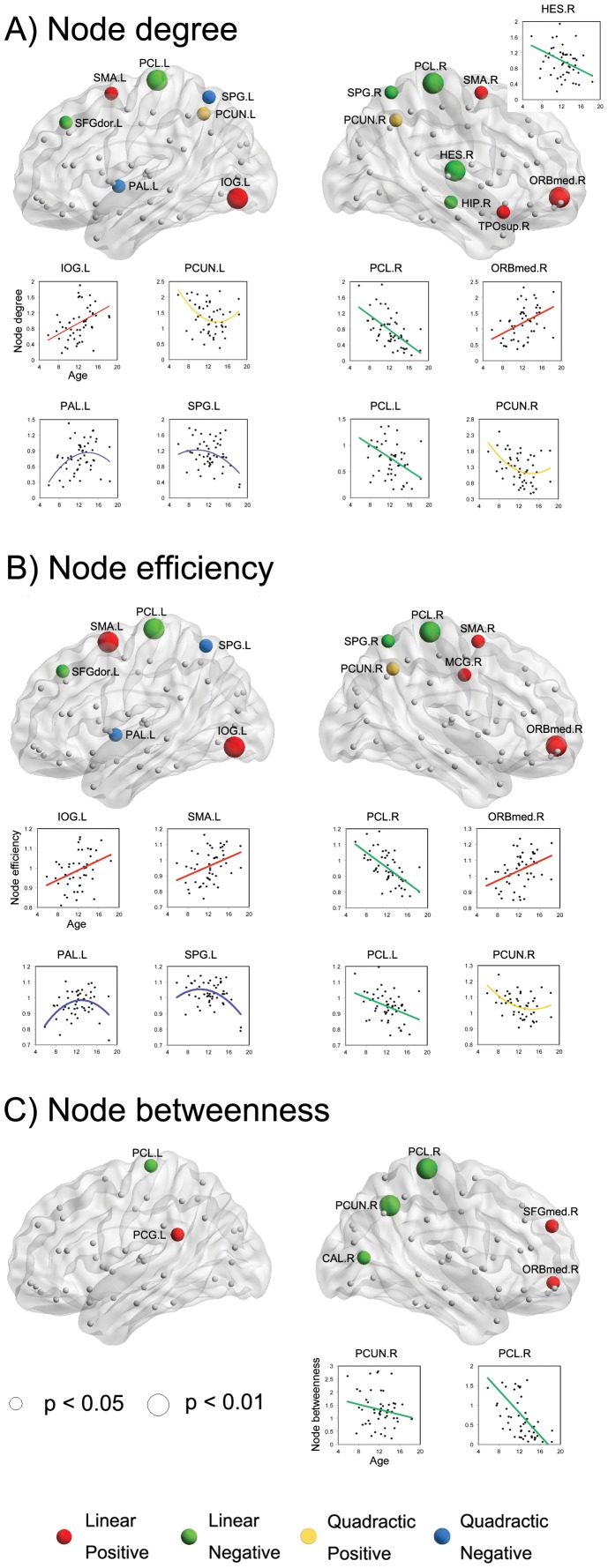
Effect of age on regional nodal properties. Significant linear positive, linear negative, quadratic positive, and quadratic negative correlations are indicated by red, green, yellow, and blue spheres, respectively. The significances of p<0.05 and p<0.01(uncorrected) are shown by spheres in small and big size, respectively. For a description of the abbreviations, see Table S1.

The presence of linear and quadratic age-related changes in the summary regional nodal parameters was examined using a GLM analysis. We identified the brain regions showing significant age-related changes (*p*<0.05 or *p*<0.01, uncorrected) in the regional nodal parameters (Figure 3). Linear age-related increases in the node degree were found in 5 brain regions, including the bilateral supplementary motor area (SMA), left inferior occipital gyrus (IOG), right ORBmed, and right superior temporal pole (TPOsup) (Figure 3A, red regions). Linear age-related decreases in the node degree were found in 6 brain regions, including the bilateral paracentral lobule (PCL), left dorsal superior frontal gyrus (SFGdor), right superior parietal gyrus (SPG), right heschl gyrus (HES), and right hippocampus (HIP) (Figure 3A, green regions). A positive quadratic (i.e., U-shaped) developmental trajectory of the node degree was observed in the bilateral PCUN (Figure 3A, yellow regions). A negative quadratic (i.e., inverted U-shaped) correlation between age and the node degree was found in the left pallidum (PAL) and left SPG (Figure 3A, blue regions). Similarly, linear age-related increases in the node efficiency were found in 5 brain regions, including the bilateral SMA, left IOG, right ORBmed, and right middle cingulate gyrus (MCG) (Figure 3B, red regions). Linear age-related decreases in the node efficiency were found in 4 brain regions, including the bilateral PCL, left SFGdor, and right SPG (Figure 3B, green regions). A positive quadratic developmental trajectory of node efficiency was observed in the right PCUN (Figure 3B, yellow regions). A negative quadratic correlation between age and the node efficiency was found in the left PAL and left SPG (Figure 3B, blue regions). Moreover, linear age-related increases in the node betweenness were found in 3 brain regions, including the left PCG, right ORBmed, and right SFGmed (Figure 3C, red regions). Linear age-related decreases in the node betweenness were found in 4 brain regions, including the bilateral PCL, right PCUN, and right calcarine cortex (CAL) (Figure 3C, green regions).

**Figure 4:**
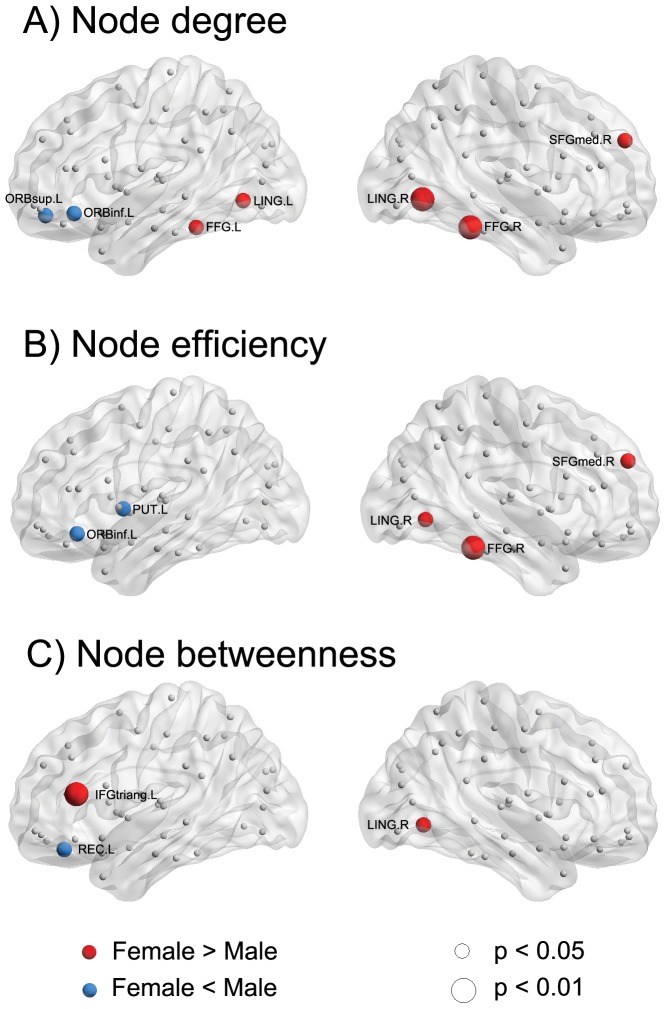
Effect of sex on regional nodal properties. The significant higher values of regional nodal parameters in female and male groups are shown in red and blue, respectively. The significances of p<0.05 and p<0.01 (uncorrected) are indicated by spheres in small and big size, respectively. For a description of the abbreviations, see Table S1.

We also found significant sex-related differences (*p*<0.05 or *p*<0.01, uncorrected) in the regional nodal parameters (Figure 4). The girls showed higher values in the node degree of the bilateral fusiform gyrus (FFG), bilateral lingual gyrus (LING), and right SFGmed (Figure 4A, red regions). The boys showed higher values in the node degree of the left superior orbitofrontal cortex (ORBsup) and left inferior orbitofrontal cortex (ORBinf) (Figure 4A, blue regions). The girls showed higher values in the node efficiency of the right FFG, right LING, and right SFGmed (Figure 4B, red regions). The boys showed higher values in the node efficiency of the left putamen (PUT) and left ORBinf (Figure 4B, blue regions). The girls showed higher values in the node betweenness of the left triangular part of the inferior frontal gyrus (IFGtriang) and right LING (Figure 4C, red regions), whereas the boys showed higher values in the node efficiency in the left rectus gyrus (REC) (Figure 4C, blue regions).

**Figure 5:**
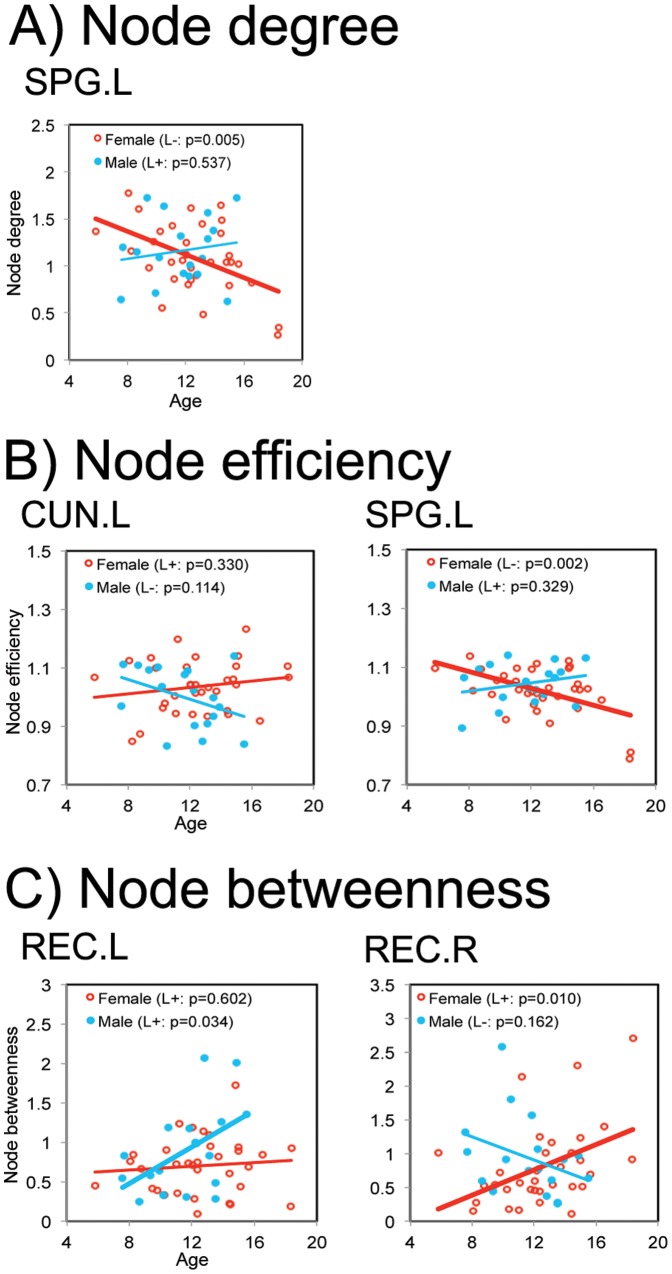
Age-by-sex interaction on regional nodal properties. The significant age-by-sex interactions on regional nodal parameters are shown. The correlation between age and regional nodal parameters are shown in female and male groups, respectively. For a description of the abbreviations, see Table S1.

Significant age-by-sex interactions (*p*<0.05, uncorrected) were also found in several brain regions (Figure 5). Further, the Pearson's correlation analysis revealed the correlations between age and the regional nodal parameters in each sex group. A significant negative correlation with age (*p* = 0.005) in the node degree in the left SPG was found in the girls, whereas a positive correlation with age (*p* = 0.537) was found in the boys (Figure 5A). The girls showed positive correlation with age (*p* = 0.330) in the node efficiency in the left cuneus (CUN), whereas the boys showed negative correlation with age (*p* = 0.114) (Figure 5B, left). A significant negative correlation with age (*p* = 0.002) in the node efficiency in the left SPG was found in the girls, whereas the boys showed positive correlation with age (*p* = 0.329) (Figure 5B, right). A significant positive correlation with age (*p* = 0.034) in the node betweenness in the left REC was found in the boys, whereas the girls showed no change with age (*p* = 0.602) (Figure 5C, left). The girls showed significant positive correlation with age (*p* = 0.010) in the node efficiency in the right REC, whereas the boys showed negative correlation with age (*p* = 0.162) (Figure 5C, right).

### IQ-related differences

**Figure 6:**
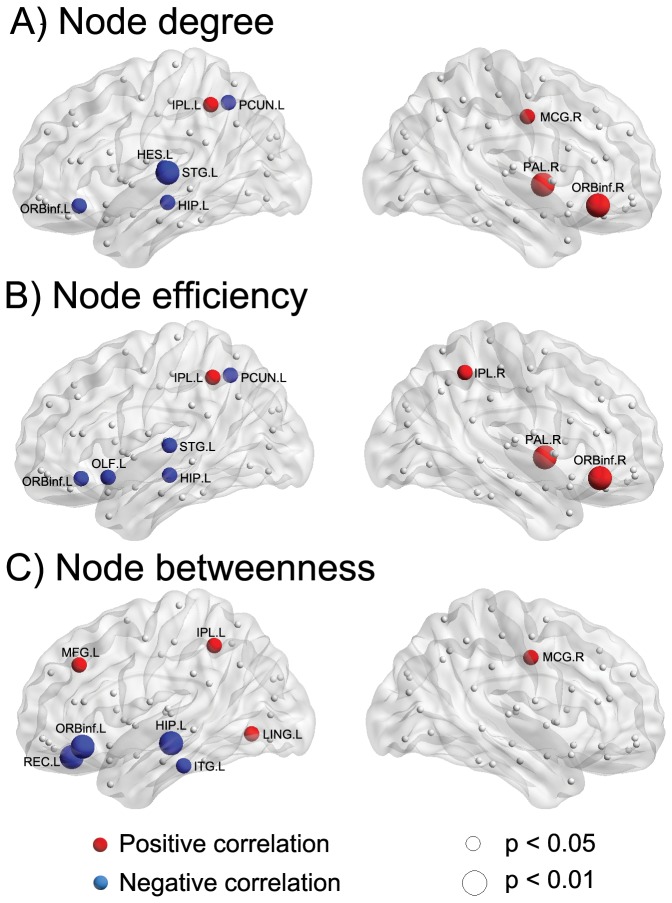
Effect of IQ on regional nodal properties. The significant positive and negative correlation between IQ and regional nodal parameters are shown in red and blue, respectively. The significances of p<0.05 and p<0.01 (uncorrected) are indicated by spheres in small and big size, respectively. For a description of the abbreviations, see Table S1.

We found no significant IQ-related difference in the summary global network parameters (Table 1). However, there were significant correlations between IQ and the regional node parameters after regressing out the effects of age, sex, and age-by-sex interaction (Figure 6). A significant positive correlation between IQ and the node degree was found in the left inferior parietal lobule (IPL), right MCG, right PAL, and ORBinf (Figure 6A, red regions). A significant negative correlation between IQ and node efficiency was found in the left PCUN, left HES, left STG, left ORBinf, and left HIP (Figure 6A, blue regions). A significant positive correlation between IQ and the node efficiency was found in the bilateral IPL, right PAL, and ORBinf (Figure 6B, red regions). A significant negative correlation between IQ and the node efficiency was found in the left PCUN, left STG, left ORBinf, left HIP, and left olfactory (OLF) (Figure 6B, blue regions). A significant positive correlation between IQ and the node betweenness was found in the left IPL, left MFG, left LING, and right MCG (Figure 6C, red regions). A significant negative correlation between IQ and the node betweenness was found in the left REC, left ORBinf, left HIP, and left inferior temporal gyrus (ITG) (Figure 6C, blue regions).

## Discussion

The present study examined the topological organization of the functional brain networks derived from rs-fMRI in healthy children and quantitatively analyzed the effects of age, sex, and IQ on the network properties at both the global and regional levels. The main findings were as follows: (1) an economical small-world organization was found in the functional brain networks of all healthy children; (2) the age-related increases in the local efficiency of the whole networks contributed to the development of modularized information processing of functional systems; (3) the boys showed a higher global efficiency compared with the girls, supporting a more optimal configuration in the boys for parallel information transfer; (4) many brain regions primarily in the frontal, parietal, and occipital lobes were profoundly affected by age, sex, and their interaction, indicating that girls and boys showed distinct developmental patterns of the functional brain networks; and (5) the regional nodal parameters positively correlated with IQ were found in several brain regions related to the attention system, whereas those negatively correlated with IQ were found in various brain regions primarily involved in the default mode, emotion, and language systems.

### Economic small-world organization

We demonstrated the key properties of economical small-world organization in the functional brain networks in all healthy children. An economical small-world network can provide a topological substrate for both locally specialized or segregated processing in the neighborhoods of highly clustered nodes and globally distributed or integrated processing on a highly efficient network with a short characteristic path length [36], [52], [53], [54]. Recent studies on structural and functional brain networks indicate that the economical small-world organization is established during a critical time period of brain development, from 2 or 3 weeks to 39 weeks of age [55], [56], [57], [58]. Thus, our results provided further support for the previous findings that the functional brain networks display small-world properties in children and young adults, indicating an efficient network structure throughout the developmental process [59], [60].

Moreover, we employed three parameters to examine the regional nodal properties in 90 brain regions. We identified 21 global hubs with higher values in either regional nodal parameter that play a vital role in the global information integration between different parts of the network. The global hubs were predominately located in the prefrontal and parietal lobes, providing a potential explanation for their well-documented activation by many cognitive functions [3], [48]. The global hubs identified in this study have also been recognized as global hubs by previous studies on structural and functional brain networks [10], [11], [37], [52], [61], [62], [63], [64], [65]. Interestingly, several global hubs (bilateral SFGmed and left PCUN) have been recently indicated as the core of a rich-club organization, playing a central role in information integration and in conferring robustness to its structural core [66].

### Effects of age and sex on global network properties

Recent advances in MRI technology have enabled precise measurements of functional interactions between brain regions and have provided significant insights into human brain development [7], [8], [67]. In this study, we applied a graph theoretical analysis on the functional connectivities in the whole brains of healthy children and found that several global network parameters (e.g., *NC*, *SW*, and *LE*) showed significant increases with age. The age-related differences in the global network properties were mainly attributed to the increases in the clustering or local efficiency with development, whereas the global efficiency was not significantly related to age. The higher local efficiency has been suggested to be associated with the modularized information processing among topologically nearby regions [1], [3]. Functionally specialized brain regions typically show high clustering due to an abundance of connections to other areas with the same functional specialization (e.g., visual processing) and in the same anatomical neighborhood (e.g., the occipital cortex) [68]. Thus, our results supported the notion that the functionally related regions or brain systems (e.g., the default mode system and the control system) emerge during development in childhood and adolescence [69], [70]. These results were also consistent with previous findings that the organization of multiple functional networks shifts from a local anatomical emphasis in children to a more distributed architecture in young adults, indicating the maturation process of the functional systems [59]. Our findings also provided some implications for understanding the inverse trajectories with advancing age (e.g., the decreased local efficiency) in functional [36] and structural [10], [37] brain networks. Moreover, the stable global efficiency in children might be associated with the fact that the path length of the functional brain networks in early childhood was already as short as those of random networks, as previous studies indicated [59], [60]. Furthermore, our results of the age-related increase in small-worldness indicated that the topological organization of the functional brain networks developed in healthy children to promote an optimal balance between segregation and integration for robust and dynamic information processing in the human brain [71]. Notably, statistical comparisons were not made in the small-world properties by either Fair et al. (2009) or Fransson et al. (2011), and Supekar et al. (2009) made comparisons at a single edge density (cost threshold, *t* = 0.54). Thus, this study, which statistically analyzed the summary global network parameters calculated from the small-world regime (0.2≤*t*≤0.35), provided evidence for the age-related differences in the economical small-world properties of the whole-brain functional networks in healthy children.

The functional connectivity derived from functional MRI is also modulated by gender [72]. In this study, we observed sex-related differences in the functional brain networks constructed from functional connectivity in healthy children. The boys showed significantly higher values in the global efficiency (*p* = 0.027), whereas the girls showed marginally significantly higher values in the clustering coefficient (*p* = 0.055). A recent study on functional brain networks in young adults showed a gender-by-hemisphere interaction that men had a higher normalized clustering coefficient in the right hemispheric network but a lower clustering coefficient in the left hemispheric network, indicating a finding different from this study, most likely due to the lack of inter-hemisphere connections [11]. However, our results were consistent with a previous study on the structural brain networks constructed from DTI with young adults in which females have higher local efficiencies than males [9]. Thus, we speculated that the functional brain networks in the boys showed a more optimal configuration for globally distributed processing, whereas those in the girls took an advantage of locally specialized processing. Integrated processes (e.g., the executive functions) would benefit from the global efficiency of information transfer across the network as a whole, whereas segregated processes (e.g., aspects of visual-input analysis) would benefit from highly clustered connections between topological neighbors [68]. Moreover, a high global efficiency assures effective integrity or rapid transfers of information between and across remote regions that are believed to constitute the basis of cognitive process [54]. Sex differences in the cognitive functions are well-known to become more pronounced during childhood and adolescence [73]. Therefore, our findings of a sex difference in the functional brain networks possibly underlied the sex-related cognitive differences in children.

### Effects of age and sex on regional nodal properties

The age-related changes in the regional nodal properties were predominately found in the frontal and parietal lobes. Age-related increases were found in several frontal brain regions (e.g., SFGmed.R, ORBmed.R, SMA.L, SMA.R, and MCG.R), which appear to be those of the last brain regions to mature and related to the increasing cognitive capacity during childhood [74]. Moreover, several key brain regions (e.g., PCG.L, SFGmed.R, and ORBmed.R) of the default mode network (DMN) [75], [76] showed age-related increases. These findings were consistent with evidence that the DMN is only sparsely connected (i.e., fragmented) and becomes significantly more integrated during development [70]. However, two of the key DMN regions (bilateral PCUN) showed a positive quadratic (e.g., U-shaped) developmental trajectory or a linear age-related decrease in the regional nodal properties. A previous study on the structural brain networks in healthy pediatric subjects during the first years of life indicated that the right precuneus shows a quite high value in the node betweenness among all brain regions [57]. In this study, the bilateral precuneus also showed high values in the regional node parameters (identified as a global hub) across all subjects. Thus, regarding the functional connectivity, we assumed that the bilateral precuneus was likely to be developed early, preserving the high values with a slight decrease during childhood. We also found several brain regions showing a linear age-related decrease (e.g., bilateral PCL, SPG.R, SFGdor.L, HES.R, HIP.R, and CAL.R) and a negative quadratic (e.g., inverted U-shape) developmental trajectory (e.g., SPG.L and PAL.L), which are primarily related to motor, somatosensory, auditory, and visual functions. Recent studies demonstrate that the global hubs in functional brain networks are largely confined to primary sensory and motor brain regions in the infant brain [56], [58]. However, the global hubs identified in the adult brain are mainly composed of heteromodal association cortices [51], as indicated by recent studies on structural [10], [37], [63], [64], [77], [78] and functional [52], [62] brain networks. Therefore, our results suggested that the frontal brain regions associated with the higher-order cognitive functions developed during childhood, whereas the parietal, temporal, and occipital brain regions related to the primary motor/somatosensory, auditory, and visual functions tended to play a less important role in the whole brain due to their early development.

Significant sex-related differences in the regional nodal parameters were also found in various brain regions, which were primarily related to the default mode system (e.g., SFMmed.R), the language system (e.g., IFGtriang.L, REC.L, ORBinf.L, and PUT.L), and the vision system (e.g., bilateral LING and FFG). Our results were consistent with the notion that cognitive and emotional development differs between females and males, particularly in visuospatial, language, and emotion processing [73], [79], [80]. Interestingly, the identified brain regions involved in the language system were all from the left hemisphere, whereas those related to the visuospatial processing were from the right hemisphere. These findings supporting the notion that differences in laterality between males and females when processing language versus visuospatial information [81], [82].

We also identified age-by-sex interactions in several brain regions associated with the visuospatial function (e.g., CUN.L and SPG.L) [81], [83] and emotion processing (e.g., bilateral REC) [84], [85], [86]. These sex-dimorphic patterns of developmental trajectories in the brain regions may be the result of underlying sex differences in the functional maturation of these regions. Furthermore, sex differences in brain development have been well documented to potentially be related to the prevalence, course, and treatment of several neuropsychiatric disorders, such as autism, attention deficit hyperactivity disorder (ADHD), and schizophrenia [87], [88]. Recent studies on functional and structural brain networks have indicated significant changes in the regional node properties in autism [89], ADHD [41], and schizophrenia [39], [40], [90], [91]. For example, two brain regions (e.g., IFGtriang.L and REC.L) showing significant sex-related differences in this study were found to be altered in the ADHD group [41]. Therefore, investigating the sex-specific brain networks in health and neurodevelopmental disorders will be interesting to study in the future.

### IQ-related differences

Previous studies of fMRI on working memory tasks [92], verbal and non-verbal reasoning tasks [93], [94], [95], and at rest [96] have indicated that the functional interactions between multiple brain regions are strongly related to the neural basis of intelligence. In this study, we investigated the correlation between IQ and the topological properties of functional brain networks. We found no IQ-related change in the global network properties. However, recent studies showed that intelligence is highly correlated with both the global and local efficiencies of structural and functional brain networks [15], [16], [91]. Several factors might contribute to the discrepancies between the present study and previous studies. First, the subjects included in this study were healthy children, whereas the previous studies all involved adults. The IQ-related differences in the network properties would be affected by the developmental trajectories of the functional connectivity between sexes [97]. Therefore, separating age- and sex-specific groups to investigate the correlation between brain network properties and intelligence would be useful. Second, although the similar parcellation of the whole brain used in this study and the previous studies on the structural brain networks derived from DTI [15], [91], different MRI modalities may affect the organization of brain networks. Third, the previous studies on the functional brain network using rs-fMRI [16] is a voxel-wise network study including approximately 9500 voxels, which may result in the differences in network properties due to the different network size [91], [98], [99]. Finally, we investigated the global network parameters using the average value across the small-world regime, which might rule out some significances at a certain cost threshold as observed in the previous studies.

The IQ-related differences in the regional nodal parameters were found in the brain regions predominately in the frontal, parietal, and temporal lobes. Consistent with the parieto-frontal integration theory (P-FIT) [100], several brain regions identified in this study (e.g., STG.L, ITG.L, HIP.L, and LING.L) were related to the basic sensory/perceptual processing of cognitively salient information predominantly through the combination of auditory and visual means; the interaction of parieto-frontal brain regions (e.g., bilateral IPL, bilateral ORBinf, PCUN.L, MFG.L, MCG.R, REC.L, OLF.L) underpins higher cognitive functions. Thus, our results provided further evidence for the notion proposed by the P-FIT model that variations in a distributed brain network predict individual differences in intelligence. The distributed brain network closely related to intelligence was also indicated by previous studies on functional connectivity [96], anatomical brain networks [15], and functional brain networks [16]. Moreover, several brain regions mainly involved in the attention system (e.g., the bilateral IPL, MFG.L, MCG.R, and ORBinf.R) showed a positive correlation with IQ, whereas the brain regions negatively correlated with IQ were primarily associated with the default mode system (e.g., PCUN.L), emotion system (e.g., ORBinf.L, OLF.L, REC.L, and HIP.L), and language system (e.g., STG.L, HES.L, and ITG.L). One interpretation of the opposed correlation with IQ between the attention system and the default mode system might be recent findings of anticorrelations between the default and attention subsystems [34], [101]. However, a previous study revealed that several key brain regions involved in the default mode system (e.g., medial prefrontal cortex, bilateral inferior parietal cortex, and precuneus/posterior cingulate regions) showed strong negative correlation between the full-scale IQ and individual normalized path length, indicating high node efficiency [16]. Several issues might contribute to the different findings of this study, such as preprocessing methods of rs-fMRI (see the discussion in the methodology issues) and the factors that we discussed before (e.g., subjects, network size, and threshold strategy). Together, our findings indicated that the brain regions related to the attention, default mode, emotion, and language systems were important predictors for the differences in intelligence in children with normal development.

### Methodological issue

There are several issues that should be addressed. Two recent studies indicate that head motion produces substantial changes in rs-fMRI analysis despite compensatory spatial registration and regression of motion estimates; these changes had significant and systematic effects on functional network measures [102], [103]. To evaluate that the main results of this study were not greatly affected by head motion, we (1) calculated 2 indices of data quality introduced by [102]: Frame-wise displacement (FD) and the RMS variance of the temporal derivative of time courses (DVARS); (2) interpolated time points where FD >0.5 mm and DVARS >0.5 with nearest neighbor using cubic spline interpolation [104]; (3) analyzed all global and regional properties of functional brain networks. With and without interpolation of motion artifact, the analysis of global network properties showed same results (e.g., significant age-related increase on local efficiency; higher local efficiency but lower global efficiency in girl). Moreover, regional nodal properties calculated from functional connectivity before and after interpolation also showed high correlations (node degree, *r* = 0.9857; node efficiency, *r* = 0.9776; node betweenness, *r* = 0.8903). Together, the main results reported are likely not attributable to the effects of head motion.

The effects of age, sex, and IQ on the topological organization of brain networks in healthy children should be examined carefully and precisely. Significant interactions of age-by-sex, sex-by-IQ, or age-by-sex-by-IQ have been identified in previous studies of children on functional connectivity [97], [105], [106], anatomical connectivity [107], brain perfusion [23], and brain structure [50], [108], [109], [110], [111], [112]. Moreover, the significances in the regional node properties found in this study were at the level of *p*<0.05 without multiple comparisons correction; an even higher level of significance might be able to be achieved by including more subjects. Therefore, a large number of subjects will be useful for the investigation of the effects of age, sex, and IQ on brain network properties in future studies. Furthermore, sexually dimorphic trajectories in the brain structure [108], IQ-related trajectories of cortical development [110], and the established topological organization of brain anatomical networks in early brain development [55] have been confirmed by longitudinal designs. Therefore, although this study was a cross-sectional study, a longitudinal analysis would also be helpful to examine the developmental trajectory of brain networks in healthy children and its relationships with sex and IQ. Finally, investigating the topological organization of the human brain networks during development in combination with functional and structural studies using multi-modality MRI, such as DTI, structural MRI, and rs-fMRI, is also important.

## Conclusions

In conclusion, we observed an economical small-world organization in the functional brain networks derived from rs-fMRI in healthy children. Our results indicated significant effects of age, sex, and IQ on both global and regional nodal properties. These findings may help elucidate normal brain maturation, which merits further investigations that include a larger number of subjects, apply a longitudinal design, and combine multimodality MRI data. Our study provided insights into the maturation mechanism of the functional brain systems in healthy children from a network perspective.

## Supporting Information

Table S1 **Regions of interest included in AAL-atlas.**
(DOC)

Table S2 **Global hubs in functional brain networks using binary network analysis.**
(DOC)

Table S3 **Global hubs in functional brain networks using weighted network analysis.**
(DOC)

Table S4 **The effects of age, sex, and IQ on global network properties using weighted network analysis.**
(DOC)

Table S5 **Effect of age on regional nodal properties using weighted network analysis.**
(DOC)

Table S6 **Effect of sex on regional nodal properties using weighted network analysis.**
(DOC)

Table S7 **Age-by-sex interaction on regional nodal properties using weighted network analysis.**
(DOC)

Table S8 **Pearson's correlation between age and regional nodal properties in each sex group using weighted network analysis.**
(DOC)

Table S9 **Effect of IQ on regional nodal properties using weighted network.**
(DOC)

Text S1 **Weighted network analysis.**
(DOC)

Text S2 **Results of weighted network analysis.**
(DOC)
