# Supplementary material for: Topological Organization of Functional Brain Networks in Healthy Children: Differences in Relation to Age, Sex, and Intelligence
Source: PLoS One. 2013 Feb 4;8(2):e55347. doi: 10.1371/journal.pone.0055347 (PMC3563524; doi:10.1371/journal.pone.0055347)
Supplement: Table S1 — Regions of interest included in AAL-atlas. (DOC) [file pone.0055347.s001.doc]

**Table S1 Regions of interest included in AAL-atlas**

| **Lobes** | **Regions** | **Abbreviations** | **Lobes** | **Regions** | **Abbreviations** |
| --- | --- | --- | --- | --- | --- |
| **Frontal** | Precentral gyrus | PreCG | **Temporal** | Hippocampus | HIP |
|  | Superior frontal gyrus  (dorsal) | SFGdor |  | Parahippocampal gyrus | PHG |
|  | Orbitofrontal cortex  (superior) | ORBsup |  | Amygdala | AMYG |
|  | Middle frontal gyrus | MFG |  | Fusiform gyrus | FFG |
|  | Orbitofrontal cortex  (middle) | ORBmid |  | Heschl gyrus | HES |
|  | Inferior frontal gyrus  (opercular) | IFGoperc |  | Superior temporal gyrus | STG |
|  | Inferior frontal gyrus  (triangular) | IFGtriang |  | Temporal pole (superior) | TPOsup |
|  | Orbitofrontal cortex  (inferior) | ORBinf |  | Middle temporal gyrus | MTG |
|  | Rolandic operculum | ROL |  | Temporal pole (middle) | TPOmid |
|  | Supplementary motor  area | SMA |  | Inferior temporal gyrus | ITG |
|  | Olfactory | OLF | **Occipital** | Calcarine cortex | CAL |
|  | Superior frontal gyrus  (medial) | SFGmed |  | Cuneus | CUN |
|  | Orbitofrontal cortex  (medial) | ORBmed |  | Lingual gyrus | LING |
|  | Rectus gyrus | REC |  | Superior occipital gyrus | SOG |
|  | Anterior cingulate gyrus | ACG |  | Middle occipital gyrus | MOG |
|  | Middler cingulate gyrus | MCG |  | Inferior occipital gyrus | IOG |
|  |  |  |  |  |  |
| **Parietal** | Posterior cingulate gyrus | PCG | **Subcortical** | Caudate | CAU |
|  | Postcentral gyrus | PoCG |  | Putamen | PUT |
|  | Superior parietal gyrus | SPG |  | Pallidum | PAL |
|  | Inferior parietal lobule | IPL |  | Insula | INS |
|  | Supramarginal gyrus | SMG |  | Thalamus | THA |
|  | Angular gyrus | ANG |  |  |  |
|  | Precuneus | PCUN |  |  |  |
|  | Paracentral lobule | PCL |  |  |  |
