# Supplementary material for: Topological Organization of Functional Brain Networks in Healthy Children: Differences in Relation to Age, Sex, and Intelligence
Source: PLoS One. 2013 Feb 4;8(2):e55347. doi: 10.1371/journal.pone.0055347 (PMC3563524; doi:10.1371/journal.pone.0055347)
Supplement: Table S2 — Global hubs in functional brain networks using binary network analysis. (DOC) [file pone.0055347.s002.doc]

**Table S2 Global hubs in functional brain networks using binary network analysis**

| **Lobe** | **Class** | **Brain regions** | **Node degree** | | **Node efficiency** | | **Node betweenness** | |
| --- | --- | --- | --- | --- | --- | --- | --- | --- |
|  |  |  | Mean | SD | Mean | SD | Mean | SD |
| Frontal | Association | MFG.R | 1.090 | 0.298 | 1.025 | 0.072 | **1.285** | **0.627** |
| Frontal | Association | ROL.L | **1.225** | **0.352** | **1.056** | **0.084** | 1.086 | 0.561 |
| Frontal | Association | ROL.R | **1.238** | **0.319** | **1.059** | **0.077** | 1.165 | 0.621 |
| Frontal | Association | SFGmed.L | **1.222** | **0.324** | **1.057** | **0.076** | **1.368** | **0.593** |
| Frontal | Association | SFGmed.R | **1.180** | **0.326** | **1.046** | **0.079** | 1.205 | 0.599 |
| Frontal | Paralimbic | ORBmed.R | 1.146 | 0.418 | 1.036 | 0.101 | **1.248** | **0.677** |
| Subcortical | Paralimbic | INS.L | **1.307** | **0.242** | **1.075** | **0.054** | **1.555** | **0.557** |
| Subcortical | Paralimbic | INS.R | **1.422** | **0.284** | **1.102** | **0.064** | **1.801** | **0.665** |
| Frontal | Paralimbic | ACG.L | **1.183** | **0.347** | **1.045** | **0.084** | **1.278** | **0.584** |
| Frontal | Paralimbic | ACG.R | **1.215** | **0.340** | **1.053** | **0.081** | **1.332** | **0.677** |
| Parietal | Paralimbic | PCG.L | **1.204** | **0.379** | **1.051** | **0.090** | 1.152 | 0.531 |
| Occipital | Association | SOG.R | **1.232** | **0.321** | **1.058** | **0.075** | **1.281** | **0.616** |
| Parietal | Primary | PoCG.L | **1.231** | **0.376** | **1.056** | **0.092** | **1.234** | **0.660** |
| Parietal | Primary | PoCG.R | **1.192** | **0.356** | **1.049** | **0.085** | **1.307** | **0.773** |
| Parietal | Association | SMG.R | **1.174** | **0.359** | **1.044** | **0.085** | **1.360** | **0.821** |
| Parietal | Association | ANG.L | **1.173** | **0.351** | **1.045** | **0.085** | **1.261** | **0.576** |
| Parietal | Association | ANG.R | **1.217** | **0.317** | **1.055** | **0.074** | **1.311** | **0.622** |
| Parietal | Association | PCUN.L | **1.289** | **0.358** | **1.070** | **0.085** | **1.327** | **0.652** |
| Parietal | Association | PCUN.R | **1.190** | **0.317** | **1.049** | **0.073** | 1.177 | 0.526 |
| Temporal | Association | STG.L | **1.319** | **0.321** | **1.076** | **0.078** | **1.316** | **0.608** |
| Temporal | Association | STG.R | **1.234** | **0.343** | **1.059** | **0.081** | **1.225** | **0.652** |

Global hubs are defined as the brain regions with higher values (>mean + SD, indicated by bold characters) in any of the regional nodal parameters.
