# Supplementary material for: Topological Organization of Functional Brain Networks in Healthy Children: Differences in Relation to Age, Sex, and Intelligence
Source: PLoS One. 2013 Feb 4;8(2):e55347. doi: 10.1371/journal.pone.0055347 (PMC3563524; doi:10.1371/journal.pone.0055347)
Supplement: Table S3 — Global hubs in functional brain networks using weighted network analysis. (DOC) [file pone.0055347.s003.doc]

**Table S3 Global hubs in functional brain networks using weighted network analysis**

| **Lobe** | **Class** | **Brain regions** | **Node strength** | | **Node efficiency** | | **Node betweenness** | |
| --- | --- | --- | --- | --- | --- | --- | --- | --- |
|  |  |  | Mean | SD | Mean | SD | Mean | SD |
| Frontal | Association | SFGdor.R | 0.441 | 0.039 | 0.299 | 0.041 | **1.202** | **0.545** |
| Frontal | Association | MFG.R | 0.453 | 0.037 | 0.304 | 0.031 | **1.215** | **0.564** |
| Frontal | Association | ROL.L | **0.476** | **0.049** | **0.322** | **0.045** | 0.984 | 0.474 |
| Frontal | Association | ROL.R | **0.478** | **0.048** | **0.325** | **0.043** | 1.018 | 0.564 |
| Frontal | Association | SFGmed.L | 0.463 | 0.041 | **0.319** | **0.039** | **1.292** | **0.627** |
| Frontal | Association | SFGmed.R | 0.462 | 0.044 | **0.315** | **0.042** | 1.151 | 0.562 |
| Subcortical | Paralimbic | INS.L | **0.468** | **0.037** | **0.326** | **0.028** | **1.371** | **0.533** |
| Subcortical | Paralimbic | INS.R | **0.480** | **0.043** | **0.340** | **0.032** | **1.398** | **0.601** |
| Frontal | Paralimbic | ACG.L | 0.457 | 0.043 | 0.312 | 0.040 | **1.319** | **0.527** |
| Frontal | Paralimbic | ACG.R | 0.462 | 0.043 | **0.316** | **0.040** | **1.235** | **0.574** |
| Frontal | Paralimbic | MCG.R | 0.445 | 0.042 | 0.300 | 0.039 | **1.169** | **0.632** |
| Parietal | Paralimbic | PCG.L | 0.464 | 0.046 | **0.317** | **0.045** | 1.067 | 0.482 |
| Occipital | Primary | CAL.L | **0.473** | **0.045** | 0.293 | 0.038 | 0.763 | 0.505 |
| Occipital | Primary | CAL.R | **0.476** | **0.049** | 0.294 | 0.045 | 0.800 | 0.486 |
| Occipital | Association | CUN.L | 0.463 | 0.047 | 0.307 | 0.041 | **1.187** | **0.797** |
| Occipital | Association | CUN.R | **0.468** | **0.036** | 0.302 | 0.030 | 0.967 | 0.507 |
| Occipital | Association | LING.L | **0.475** | **0.053** | 0.300 | 0.048 | 0.836 | 0.432 |
| Occipital | Association | LING.R | **0.482** | **0.054** | 0.309 | 0.051 | 0.801 | 0.380 |
| Occipital | Association | SOG.L | **0.472** | **0.051** | 0.312 | 0.044 | 0.994 | 0.598 |
| Occipital | Association | SOG.R | **0.470** | **0.054** | **0.320** | **0.044** | 1.110 | 0.522 |
| Parietal | Primary | PoCG.L | **0.472** | **0.050** | **0.321** | **0.047** | 1.044 | 0.528 |
| Parietal | Primary | PoCG.R | 0.464 | 0.045 | **0.315** | **0.044** | **1.210** | **0.693** |
| Parietal | Association | SPG.L | 0.455 | 0.041 | 0.307 | 0.036 | 1.116 | 0.505 |
| Parietal | Association | SPG.R | 0.450 | 0.040 | 0.304 | 0.040 | **1.175** | **0.716** |
| Parietal | Association | SMG.R | 0.454 | 0.043 | 0.312 | 0.040 | **1.255** | **0.754** |
| Parietal | Association | ANG.R | 0.459 | 0.042 | **0.317** | **0.037** | **1.217** | **0.597** |
| Parietal | Association | PCUN.L | 0.466 | 0.044 | **0.323** | **0.041** | **1.190** | **0.569** |
| Parietal | Association | PCUN.R | 0.466 | 0.040 | **0.317** | **0.035** | 1.077 | 0.466 |
| Temporal | Association | STG.L | **0.480** | **0.043** | **0.329** | **0.038** | 1.150 | 0.647 |
| Temporal | Association | STG.R | **0.475** | **0.048** | **0.323** | **0.043** | 1.085 | 0.471 |

Global hubs are defined as the brain regions with higher values (>mean + SD, indicated by bold characters) in any of the regional nodal parameters.
