# Supplementary material for: Topological Organization of Functional Brain Networks in Healthy Children: Differences in Relation to Age, Sex, and Intelligence
Source: PLoS One. 2013 Feb 4;8(2):e55347. doi: 10.1371/journal.pone.0055347 (PMC3563524; doi:10.1371/journal.pone.0055347)
Supplement: Table S4 — The effects of age, sex, and IQ on global network properties using weighted network analysis. (DOC) [file pone.0055347.s004.doc]

**Table S4 The effects of age, sex, and IQ on global network properties using weighted network analysis**

|  | Age effect*^a^* | | |  | Sex effect*^b^* | | |  | Age-sex interaction*^b^* | |  | IQ effect*^c^* | |
| --- | --- | --- | --- | --- | --- | --- | --- | --- | --- | --- | --- | --- | --- |
|  | T-score | *p*-value | Model |  | T-score | *p*-value | Model |  | T-score | *p*-value |  | *r* | *p*-value |
| *C* | 1.935 | 0.059 | L+ |  | -1.979 | 0.054 | F>M |  | -0.492 | 0.625 |  | -0.052 | 0.715 |
| *L* | 0.674 | 0.503 |  |  | 1.661 | 0.103 |  |  | 0.844 | 0.403 |  | -0.032 | 0.825 |
| *NC* | **2.176** | **0.035** | **L+** |  | 0.192 | 0.849 |  |  | 1.444 | 0.155 |  | 0.058 | 0.686 |
| *NL* | 0.395 | 0.695 |  |  | -1.946 | 0.058 | F>M |  | 1.046 | 0.301 |  | -0.200 | 0.159 |
| *SW* | 1.990 | 0.052 | L+ |  | 0.537 | 0.594 |  |  | 1.199 | 0.237 |  | 0.093 | 0.515 |
| *LE* | **2.572** | **0.013** | **L+** |  | -1.795 | 0.079 |  |  | -0.614 | 0.542 |  | 0.025 | 0.860 |
| *GE* | -0.824 | 0.414 |  |  | -1.676 | 0.100 |  |  | -0.822 | 0.415 |  | 0.048 | 0.736 |

*^a^* Two multiply linear regressions that modeled age and age^2^ as predictors, along with sex as a covariate; the best model was determined by AIC.

*^b^* A multiply linear regression that modeled age, sex, and age-sex interaction.

*^c^* Pearson’s correlation analysis between IQ and global network properties, each of which was regressed by a multiply linear regression that modeled age, sex, and age-sex interaction.

L+: Linear regression model showing significant positive correlation. L-: Linear regression model showing significant negative correlation. Significances are set at *p*<0.05 and shown by bold characters.

L+: Linear regression model showing marginally significant positive correlation.

F>M: female shows marginally significantly higher values than male.

*C*, clustering coefficient; *L*, characteristic path length; *NC*, normalized clustering coefficient; *NL*, normalized characteristic path length; *SW*, small-worldness; *LE*, local efficiency; *GE*, global efficiency.
