# Supplementary material for: Topological Organization of Functional Brain Networks in Healthy Children: Differences in Relation to Age, Sex, and Intelligence
Source: PLoS One. 2013 Feb 4;8(2):e55347. doi: 10.1371/journal.pone.0055347 (PMC3563524; doi:10.1371/journal.pone.0055347)
Supplement: Table S5 — Effect of age on regional nodal properties using weighted network analysis. (DOC) [file pone.0055347.s005.doc]

**Table S5 Effect of age on regional nodal properties using weighted network analysis**

|  |  |  |  | Node strength | | Node efficiency | | Node betweenness | |
| --- | --- | --- | --- | --- | --- | --- | --- | --- | --- |
|  |  |  |  | T-value | *p*-value | T-value | *p*-value | T-value | *p*-value |
| Linear positive | | |  |  |  |  |  |  |  |
|  | Frontal | Primary | PreCG.L | 2.984 | 0.004 |  |  |  |  |
|  | Frontal | Association | IFGoperc.L | 3.253 | 0.002 |  |  |  |  |
|  | Frontal | Association | IFGtriang.L | 3.292 | 0.002 |  |  |  |  |
|  | Frontal | Paralimbic | ORBinf.L | 3.312 | 0.002 |  |  |  |  |
|  | Frontal | Association | SMA.L |  |  | 3.179 | 0.003 |  |  |
|  | Frontal | Association | SMA.R |  |  | 3.138 | 0.003 |  |  |
|  | Frontal | Paralimbic | ORBmed.R | 3.859 | 0.000 | 4.460 | 0.000 |  |  |
|  | Frontal | Paralimbic | REC.L |  |  |  |  | 2.229 | 0.031 |
|  | Frontal | Paralimbic | REC.R |  |  | 2.961 | 0.005 | 2.326 | 0.024 |
|  | Frontal | Paralimbic | MCG.R | 3.105 | 0.003 | 3.220 | 0.002 |  |  |
|  | Temporal | Paralimbic | TPOsup.R | 3.601 | 0.001 | 3.034 | 0.004 |  |  |
|  | Occipital | Association | IOG.L | 3.358 | 0.002 | 3.622 | 0.001 |  |  |
|  | Subcortical | Subcortical | PAL.L | 3.992 | 0.000 | 3.128 | 0.003 | 0.834 | 0.408 |
| Linear negative |  |  |  |  |  |  |  |  |  |
|  | Parietal | Association | PCL.L |  |  |  |  | -2.377 | 0.021 |
|  | Parietal | Association | PCL.R |  |  | -3.596 | 0.001 | -4.284 | 0.000 |
|  | Subcortical | Subcortical | PAL.R |  |  |  |  | -2.075 | 0.043 |
| Quadratic positive |  |  |  |  |  |  |  |  |  |
|  | Parietal | Association | PCUN.L | 2.545 | 0.014 | 2.334 | 0.024 |  |  |
|  | Parietal | Association | PCUN.R | 2.020 | 0.049 | 2.220 | 0.031 |  |  |
|  | Temporal | Association | STG.L |  |  |  |  | 2.082 | 0.043 |
|  | Temporal | Paralimbic | PHG.R |  |  |  |  | 2.442 | 0.018 |
|  | Subcortical | Subcortical | THA.L |  |  | 2.073 | 0.044 |  |  |

Significant linear positive, linear negative, and quadratic positive correlations between regional nodal properties and age are list, respectively. The significances were set at *p*<0.05 (uncorrected).
