# Supplementary material for: Topological Organization of Functional Brain Networks in Healthy Children: Differences in Relation to Age, Sex, and Intelligence
Source: PLoS One. 2013 Feb 4;8(2):e55347. doi: 10.1371/journal.pone.0055347 (PMC3563524; doi:10.1371/journal.pone.0055347)
Supplement: Table S6 — Effect of sex on regional nodal properties using weighted network analysis. (DOC) [file pone.0055347.s006.doc]

**Table S6 Effect of sex on regional nodal properties using weighted network analysis**

|  |  |  |  | Node strength | | Node efficiency | | Node betweenness | |
| --- | --- | --- | --- | --- | --- | --- | --- | --- | --- |
|  |  |  |  | T-value | *p*-value | T-value | *p*-value | T-value | *p*-value |
| Female>Male | |  |  |  |  |  |  |  |  |
|  | Frontal | Association | IFGoperc.L | -2.417 | 0.020 |  |  |  |  |
|  | Frontal | Association | IFGtriang.L | -2.086 | 0.042 |  |  |  |  |
|  | Frontal | Association | SMA.L | -2.088 | 0.042 |  |  |  |  |
|  | Frontal | Association | SMA.R | -2.267 | 0.028 |  |  |  |  |
|  | Frontal | Association | SFGmed.R |  |  | -2.516 | 0.015 |  |  |
|  | Frontal | Paralimbic | ACG.R | -2.472 | 0.017 |  |  |  |  |
|  | Temporal | Association | MTG.R | -2.256 | 0.029 |  |  |  |  |
|  | Temporal | Paralimbic | TPOmid.R | -2.100 | 0.041 |  |  |  |  |
|  | Temporal | Paralimbic | AMYG.R | -2.307 | 0.026 |  |  |  |  |
|  | Occipital | Primary | CAL.R | -2.857 | 0.006 |  |  |  |  |
|  | Occipital | Association | CUN.L | -2.910 | 0.006 | -2.736 | 0.009 |  |  |
|  | Occipital | Association | LING.L | -2.902 | 0.006 | -2.581 | 0.013 |  |  |
|  | Occipital | Association | LING.R | -2.368 | 0.022 | -2.749 | 0.008 |  |  |
|  | Occipital | Association | IOG.L | -2.185 | 0.034 | -2.186 | 0.034 |  |  |
|  | Occipital | Association | IOG.R | -2.511 | 0.016 |  |  |  |  |
|  | Occipital | Association | FFG.L | -2.384 | 0.021 | -2.469 | 0.017 |  |  |
|  | Occipital | Association | FFG.R | -3.005 | 0.004 | -3.281 | 0.002 |  |  |

The significant higher values of regional nodal parameters in female group are list. The significances were set at *p*<0.05 (uncorrected).
