# Supplementary material for: Topological Organization of Functional Brain Networks in Healthy Children: Differences in Relation to Age, Sex, and Intelligence
Source: PLoS One. 2013 Feb 4;8(2):e55347. doi: 10.1371/journal.pone.0055347 (PMC3563524; doi:10.1371/journal.pone.0055347)
Supplement: Table S7 — Age-by-sex interaction on regional nodal properties using weighted network analysis. (DOC) [file pone.0055347.s007.doc]

**Table S7 Age-by-sex interaction on regional nodal properties using weighted network analysis**

|  |  |  |  | Node strength | | Node efficiency | | Node betweenness | |
| --- | --- | --- | --- | --- | --- | --- | --- | --- | --- |
|  |  |  |  | T-value | *p*-value | T-value | *p*-value | T-value | *p*-value |
| Positive |  |  |  |  |  |  |  |  |  |
|  | Parietal | Association | ANG.R |  |  |  |  | 3.523 | 0.001 |
|  | Parietal | Association | PCUN.L |  |  |  |  | 2.049 | 0.046 |
|  | Temporal | Paralimbic | TPOmid.R |  |  |  |  | 2.726 | 0.009 |
| Negative |  |  |  |  |  |  |  |  |  |
|  | Frontal | Paralimbic | REC.R |  |  |  |  | -2.633 | 0.011 |
|  | Temporal | Paralimbic | PHG.R |  |  |  |  | -2.174 | 0.035 |
|  | Temporal | Paralimbic | TPOsup.L | -2.120 | 0.039 |  |  |  |  |
|  | Occipital | Association | CUN.L |  |  | -2.245 | 0.030 |  |  |

The significant positive and negative age-by-sex interactions on regional nodal parameters are list, respectively. The significances were set at *p*<0.05 (uncorrected).
