# Supplementary material for: Topological Organization of Functional Brain Networks in Healthy Children: Differences in Relation to Age, Sex, and Intelligence
Source: PLoS One. 2013 Feb 4;8(2):e55347. doi: 10.1371/journal.pone.0055347 (PMC3563524; doi:10.1371/journal.pone.0055347)
Supplement: Table S8 — Pearson's correlation between age and regional nodal properties in each sex group using weighted network analysis. (DOC) [file pone.0055347.s008.doc]

**Table S8 Pearson’s correlation between age and regional nodal properties in each sex group** using weighted network analysis

|  |  |  |  | Node strength | | Node efficiency | | Node betweenness | |
| --- | --- | --- | --- | --- | --- | --- | --- | --- | --- |
|  |  |  |  | Female | *Male* | Female | *Male* | Female | *Male* |
|  |  |  |  | *r*-value (*p*-value ) | *r*-value (*p*-value ) | *r*-value (*p*-value ) | *r*-value (*p*-value ) | *r*-value (*p*-value ) | *r*-value (*p*-value ) |
| Positive |  |  |  |  |  |  |  |  |  |
|  | Parietal | Association | ANG.R |  |  |  |  | -0.037  (0.837) | **0.622**  **(0.006)** |
|  | Parietal | Association | PCUN.L |  |  |  |  | -0.174  (0.334) | 0.399  (0.101) |
|  | Temporal | Paralimbic | TPOmid.R |  |  |  |  | -0.306  (0.083) | **0.495**  **(0.037)** |
| Negative |  |  |  |  |  |  |  |  |  |
|  | Frontal | Paralimbic | REC.R |  |  |  |  | **0.434**  **(0.012)** | -0.343  (0.164) |
|  | Temporal | Paralimbic | PHG.R |  |  |  |  | 0.335  (0.057) | -0.315  (0.202) |
|  | Temporal | Paralimbic | TPOsup.L | **0.518**  **(0.002)** | -0.108  (0.669) |  |  |  |  |
|  | Occipital | Association | CUN.L |  |  | 0.316  (0.073) | -0.345  (0.160) |  |  |

Note that the brain regions included are those shown in the Table S7, showing significant age-by-sex interactions. The significant Pearson’s correlations are shown by bold characters. The significances were set at *p*<0.05 (uncorrected).
