# Supplementary material for: Topological Organization of Functional Brain Networks in Healthy Children: Differences in Relation to Age, Sex, and Intelligence
Source: PLoS One. 2013 Feb 4;8(2):e55347. doi: 10.1371/journal.pone.0055347 (PMC3563524; doi:10.1371/journal.pone.0055347)
Supplement: Table S9 — Effect of IQ on regional nodal properties using weighted network. (DOC) [file pone.0055347.s009.doc]

**Table S9 Effect of IQ on regional nodal properties** using weighted network

|  |  |  |  | Node strength | | Node efficiency | | Node betweenness | |
| --- | --- | --- | --- | --- | --- | --- | --- | --- | --- |
|  |  |  |  | *r*-value | *p*-value | *r*-value | *p*-value | *r*-value | *p*-value |
| Positive |  |  |  |  |  |  |  |  |  |
|  | Frontal | Paralimbic | ORBinf.R |  |  | 0.300 | 0.032 | 0.299 | 0.033 |
|  | Frontal | Association | MFG.L |  |  |  |  | 0.299 | 0.033 |
|  | Parietal | Association | IPL.L |  |  | 0.309 | 0.027 |  |  |
|  | Parietal | Association | IPL.R |  |  | 0.322 | 0.021 |  |  |
|  | Subcortical | Subcortical | PAL.R |  |  | 0.373 | 0.007 |  |  |
| Negative |  |  |  |  |  |  |  |  |  |
|  | Frontal | Association | SFGmed.L |  |  |  |  | -0.280 | 0.046 |
|  | Frontal | Association | IFGoperc.L |  |  |  |  | -0.320 | 0.022 |
|  | Frontal | Paralimbic | ORBinf.L |  |  |  |  | -0.453 | 0.001 |
|  | Frontal | Paralimbic | REC.L |  |  | -0.288 | 0.040 |  |  |
|  | Temporal | Association | STG.L |  |  | -0.289 | 0.040 |  |  |
|  | Temporal | Association | ITG.L |  |  |  |  | -0.335 | 0.016 |
|  | Temporal | Limbic | HIP.L |  |  |  |  | -0.348 | 0.012 |

The significant positive and negative correlations between regional nodal parameters and IQ are list, respectively. The significances were set at *p*<0.05 (uncorrected).
