# Supplementary material for: Topological Organization of Functional Brain Networks in Healthy Children: Differences in Relation to Age, Sex, and Intelligence
Source: PLoS One. 2013 Feb 4;8(2):e55347. doi: 10.1371/journal.pone.0055347 (PMC3563524; doi:10.1371/journal.pone.0055347)
Supplement: Text S1 — Weighted network analysis. (DOC) [file pone.0055347.s010.doc]

**Text S1 Weighted network analysis**

The functional connectivity between a pair of regions was defined as the Pearson’s correlation coefficient in the residual time courses. Thus, a functional connectivity matrix (or correlation matrix) (*r_ij_*, *N*×*N*) can be obtained for each subject. Each functional connectivity matrix can be converted to a weighted, undirected network *G* using a cost threshold (*t,* 0<*t*<1), which is equivalent to the ratio between the number of edges and all possible edges .

We adopted the same range of cost threshold (0.2≤ *t* ≤0.35) in the binary network analysis, which we described in the main text. Five small-world parameters (clustering coefficient, characteristic path length, normalized clustering coefficient, normalized characteristic path length, and small-worldness) and two efficiency parameters (local efficiency and global efficiency) were computed to characterize the global topological organization of the functional brain networks. Three regional nodal parameters (node degree, node efficiency, and node betweenness) were used to examine the properties of the 90 brain regions. The global network parameters and the regional nodal parameters were calculated using the Brain Connectivity Toolbox ([www.brain-connectivity-toolbox.net](http://www.brain-connectivity-toolbox.net/)). The statistical analysis was completely same to those used in the binary network analysis.

1. Latora, V. and M. Marchiori, *Efficient behavior of small-world networks.* Phys Rev Lett, 2001. **87**(19): p. 198701.
