# Supplementary material for: Topological Organization of Functional Brain Networks in Healthy Children: Differences in Relation to Age, Sex, and Intelligence
Source: PLoS One. 2013 Feb 4;8(2):e55347. doi: 10.1371/journal.pone.0055347 (PMC3563524; doi:10.1371/journal.pone.0055347)
Supplement: Text S2 — Results of weighted network analysis. (DOC) [file pone.0055347.s011.doc]

**Text S2 Results of weighted network analysis**

**Economic small-world organization**

At a global level, we identified the economic small-world organization in the functional brain networks in healthy children across the range of cost threshold (0.2≤ *t* ≤0.35) using weighted network analysis. For example, much higher values of clustering coefficient and local efficiency but similar values of characteristic path length and global efficiency were showed in the functional brain networks, compared with the matched random networks.

At the regional level, we defined 30 global hubs as the brain regions with higher values (>mean + SD) in any of the regional nodal parameters across all subjects (see supplementary Table S3). Most of these global hubs were found in the frontal, parietal, and occipital lobes and were association and paralimbic cortices. In particular, 18 of 21 global hubs identified in the binary network analysis were recognized as global hubs in the weighted analysis.

**Effects of age and sex on global network properties**

We analyzed the effects of age, sex, and age-by-sex interaction on the summary global network parameters (Table S4). Linear positive age-related changes (*p*<0.05) were only found in the normalized clustering coefficient and local efficiency. We found no significant sex differences (*p*<0.05) in global network parameters. However, the girls showed marginally significantly higher values in both clustering coefficient (*p*=0.054) and normalized characteristic path length (*p*=0.058) compared with the boys, which were similar to the results by the binary network analysis. There was no significant age-by-sex interaction in all global network parameters.

**Effects of age and sex on regional nodal properties**

The presence of linear and quadratic age-related changes in the summary regional nodal parameters was examined using a GLM analysis. We identified the brain regions showing significant age-related changes (p<0.05 or p<0.01, uncorrected) in the regional nodal parameters (Table S5). Linear age-related increases were mostly found in the frontal cortices, whereas linear age-related decreases were found in 2 parietal brain regions and 1 subcortical brain region. A positive quadratic (i.e., U-shaped) developmental trajectory was observed in the bilateral PCUN, left STG, right PHG, and left THA.

We also found significant sex-related differences (Table S6) and age-by-sex interactions (Table S7) in the regional nodal parameters (*p*<0.05 or *p*<0.01, uncorrected). Brain regions showing higher values in the girls were mostly found in the frontal, temporal, and occipital lobes. These brain regions were primarily related to the default mode system (e.g., right SFMmed and right ACG), the language system (e.g., left IFGoperc, left IFGtriang, and right MTG), and the vision system (e.g., bilateral LING, bilateral IOG, bilateral FFG, right CAL, and left CUN). Significant age-by-sex interactions were found in several brain regions related to the default mode system (e.g., right ANG and left PCUN), the emotion system (e.g., right REC, right TPOmid, and left TPOsup), and the visuospatial system (e.g., CUN.L). Further, the Pearson’s correlation analysis revealed the correlations between age and the regional nodal parameters in each sex group (Table S8). In particular, although there were some differences in the results by the binary and weighted network analysis, the correlations with age in both sexes found in the CUN.L in the node efficiency and in the REC.R in the node betweenness were similar in both analyses.

**IQ-related differences**

No significant IQ-related difference was found in the summary global network parameters (Table S4). However, we detected significant correlations between IQ and the regional node parameters after regressing out the effects of age, sex, and age-by-sex interaction (Table S9). Several brain regions mainly involved in the attention system (e.g., bilateral IPL, right ORBinf, and left MFG) showed a positive correlation with IQ, whereas the brain regions negatively correlated with IQ were primarily associated with the default mode system (e.g., left SFGmed), emotion system (e.g., left IFGoperc, left ORBinf, left REC, and left HIP), and language system (e.g., left STG and left ITG).
